# Relative biological effectiveness of 31 meV thermal neutrons in peripheral blood lymphocytes

**DOI:** 10.1093/rpd/ncae231

**Published:** 2025-03-10

**Authors:** Laura C Paterson, Fawaz Ali, Mohsen Naseri, David Perez Loureiro, Amy Festarini, Marilyne Stuart, Chad Boyer, Ronald Rogge, Christie Costello, Norma Ybarra, John Kildea, Richard B Richardson

**Affiliations:** Radiobiology and Health Branch, Canadian Nuclear Laboratories, 286 Plant Rd, Chalk River, ON K0J 1J0, Canada; Medical Physics Unit, McGill University, Montreal, QC H4A 3J1, Canada; Biology R&D Facility Branch, Canadian Nuclear Laboratories, 286 Plant Rd, Chalk River, ON K0J 1J0, Canada; Applied Physics Branch, Canadian Nuclear Laboratories, 286 Plant Rd, Chalk River, ON K0J 1J0, Canada; Applied Physics Branch, Canadian Nuclear Laboratories, 286 Plant Rd, Chalk River, ON K0J 1J0, Canada; Environment and Waste Technologies Branch, Canadian Nuclear Laboratories, 286 Plant Rd, Chalk River, ON K0J 1J0, Canada; Environment and Waste Technologies Branch, Canadian Nuclear Laboratories, 286 Plant Rd, Chalk River, ON K0J 1J0, Canada; Advanced Fuels and Reactor Physics Branch, Canadian Nuclear Laboratories, 286 Plant Rd, Chalk River, ON K0J 1J0, Canada; National Security and Critical Infrastructure Directorate, Canadian Nuclear Laboratories, 286 Plant Rd, Chalk River, ON K0J 1J0, Canada; Radiobiology and Health Branch, Canadian Nuclear Laboratories, 286 Plant Rd, Chalk River, ON K0J 1J0, Canada; Medical Physics Unit, McGill University, Montreal, QC H4A 3J1, Canada; Medical Physics Unit, McGill University, Montreal, QC H4A 3J1, Canada; Radiobiology and Health Branch, Canadian Nuclear Laboratories, 286 Plant Rd, Chalk River, ON K0J 1J0, Canada; Medical Physics Unit, McGill University, Montreal, QC H4A 3J1, Canada

## Abstract

The reported relative biological effectiveness (RBE) for thermal neutrons has a large range (5–51, for cytogenetic endpoints), which can confound radiation protection decision-making. To determine whether thermal neutron spectra can influence RBE, the RBE of reactor-derived thermal neutrons of average energy 31 meV was evaluated in human peripheral blood lymphocytes using two classical DNA double-strand break endpoints: the dicentric chromosome assay (DCA) and the cytokinesis-block micronucleus assay. Dose-response curves for 41 to 408 mGy revealed a preference for linear regression. Maximum RBE (RBE_M_) values of 6.7 ± 0.9 and 4.4 ± 0.7 were calculated for the DCA and the micronucleus assay, respectively. These 31 meV RBE_M_ values were significantly lower than our prior results for 64 meV thermal neutrons, which yielded a DCA RBE_M_ of 11.3 ± 1.6 and a micronucleus RBE_M_ of 9.0 ± 1.1. Dose-specific RBE values decreased with increasing dose for both assays. Microdosimetry simulations demonstrated similar quality factor values for both thermal neutron spectra. Dose deposition differences on the cellular scale, the difference in dose rate between irradiation configurations, or a not-yet understood phenomenon may be responsible for the RBE difference between the 31 and 64 meV thermal spectra. These findings indicate that the currently accepted radiation weighting factor *w*_R_ value of 2.5 for thermal neutrons may underestimate the radiation detriment to small or shallow tissue targets including the lens of the eye.

## Introduction

The International Commission on Radiological Protection (ICRP) prescribes radiation weighting factor (*w*_R_) values by which the absorbed dose to an organ or tissue, in Gy, can be multiplied by *w*_R_ to give the equivalent dose in Sv [1]. Low linear energy transfer (LET) photons and electrons have *w*_R_ values of unity, whereas neutron *w*_R_ values originate from two energy-dependent equations with a maximum possible value of just over 20 for fast neutrons. Low-energy thermal neutrons are assigned a *w*_R_ of 2.5 for the entirety of the thermal energy range. The *w*_R_ values are primarily based on experimental relative biological effectiveness (RBE) data, and RBE values from chromosome aberrations in human lymphocytes were given special consideration by the ICRP when deriving *w*_R_ values [1]. Therefore, a comprehensive and accurate understanding of cytogenetic thermal neutron RBE is of critical importance to radiation protection programs in environments where thermal neutrons are encountered, including space exploration [2–4], high-altitude aviation [5], radiation therapy [6], and nuclear power [7]. Accurate thermal neutron RBE assessments are also pertinent to thermal neutron components of boron neutron capture therapy [8].

In the irradiation configuration detailed here, blood contained in quartz test tubes was irradiated with thermal neutrons of mean energy 31 meV. The most common elements in blood (^1^H, ^12^C, ^14^N, and ^16^O) were considered for dosimetry purposes, as well as the ^28^Si and ^16^O present in quartz. Primary incident thermal neutrons are absorbed in soft tissue primarily via the ^14^N(n,p)^14^C and ^1^H(n,γ)^2^H capture reactions. For thermal neutrons, with a nominal energy of 25 meV, incident on a slab of International Commission on Radiation Units and Measurements (ICRU) four component soft tissue [9] of thickness 2 cm, 10 cm, and 100 cm, it is calculated from first principles that the combined percentage of incident thermal neutrons removed by the two previously listed capture reactions was 4.50%, 20.55%, and 90% respectively. These neutrons undergo capture reactions in biological targets resulting in a mixed field of secondary particles, mainly protons and photons arising from the elemental composition of the target. The secondary protons and secondary photons go on to further liberate tertiary electrons from biological tissues. The 584 ± 3 keV proton and the 41.87 keV ^14^C residual nucleus from the ^14^N(n,p)^14^C reaction [10] are responsible for ~71% of the dose delivered to the test system described here [11]. The proton has a range of ~10 μm in soft tissue and the ^14^C recoil nucleus has a range of 0.19 μm in soft tissue, resulting in local energy deposition [12]. Tertiary electrons resulting from ^1^H(n,γ)^2^H, and to a lesser extent ^16^O(n,γ)^17^O and ^28^Si(n,γ)^29^Si in the quartz test tube, are responsible for the remaining dose. Despite a significant mass fraction of carbon in blood, the dose contribution from ^12^C(n,γ)^13^C capture reactions is negligible owing to a small thermal neutron capture cross-section relative to that for ^1^H.

Consequently, in small biological targets, the total absorbed dose (*D*) can be mathematically represented as the sum of the doses of the most common elements in blood and quartz, ^1^H (*D*_1H_), ^12^C (*D*_12C_), ^14^N (*D*_14N_), ^16^O (*D*_16O_), and ^28^Si (*D*_28Si_), as denoted as in Equation 1.


(1) \begin{equation*} D={D}_{1\mathrm{H}}+{D}_{12\mathrm{C}}+{D}_{14\mathrm{N}}+{D}_{16\mathrm{O}}+{D}_{28\mathrm{Si}} \end{equation*}

This can be simplified to Equation 2 where *D*_(n,γ)_ is the sum of neutron capture reactions from ^1^H, ^16^O, and ^28^Si.


(2) \begin{equation*} D={D}_{14\mathrm{N}\left(\mathrm{n},\mathrm{p}\right)14\mathrm{C}+}{D}_{\left(\mathrm{n},\mathrm{\gamma} \right)} \end{equation*}

It should be noted that in large volumes such as the human body, the relative dose contributions from the ^14^N(n,p)^14^C and (n,γ) reactions are reversed in comparison to the test system. In a larger volume, such as the whole body, the ^1^H(n,γ)^2^H capture reaction is responsible for the majority of the dose deposition [1], resulting in a lower RBE than for the blood sample setup presented here.

Prior research has demonstrated a strong correlation between absorbed dose and the yield of radiation-induced aberrations using the dicentric chromosome assay (DCA) [13] and the micronucleus assay [14]. The DCA evaluates abnormal chromosome structures (dicentric chromosomes and centric ring chromosomes) resulting from the misrepair of DNA double-strand breaks. The cytokinesis-block micronucleus assay evaluates the mal-segregation of chromosomes during mitosis owing to DNA double-strand breaks, defects in the spindle or centromere structure, or under-condensation of chromosomes [13]. No synergistic effects between the high-LET (*D*_14N(n,p)14C_) and low-LET (n,γ) dose components are expected for either cytogenetic endpoint [15].

For better understanding of the large reported variation in thermal neutron maximum RBE at minimal doses (RBE_M_) of 5–51 [15–19], as detailed in Table 1, human lymphocytes were irradiated with two different thermal neutron spectra using the E3 beam-line at Canadian Nuclear Laboratories’ (CNL’s) National Research Universal (NRU) reactor. Ali *et al*. [11] previously published a comprehensive description of the spectra and the associated theoretical modeling of the absorbed doses from the thermal neutron capture reactions to human blood samples contained within quartz test tubes. For the first set of irradiations, previously published by Paterson *et al*. [19], a pyrolytic graphite (PG) beam-line insert produced a thermal neutron energy spectrum with an average energy of 64 meV for doses from 6 mGy to 85 mGy. These irradiations provided support for a bi-modal energy-dependent RBE distribution for neutrons.

**Table 1:** Comparison of thermal neutron RBE_M_ values generated using the DCA from peer-reviewed publications. Error on RBE values is given as SE.

Average neutron energy (meV)	Citation	Dose rate (mGy h^−1^)	RBE_M_ ± SE
Thermal	Sevan’kaev *et al.* [16]	300	10.8 ± 1.8^a^
Thermal	Wojcik *et al*. [15]	1364–5373	5.40^b^
25	Sasaki *et al*. [17]	-	51.1 ± 31.3^a^
25.3	Schmid *et al*. [18]	22500	36.4 ± 13.3
31	This study	191	6.7 ± 0.9
64	Paterson *et al*. [19]	22	11.3 ± 1.6

^a^Calculated by Schmid *et al*. [18]; ^b^2 cm water phantom used.

Here, a second set of thermal neutron irradiations is detailed using the same radiobiological methodology and again employing the NRU reactor but with a super mirror beam-line insert [11]. A continuous neutron energy spectrum was selected with a lower average energy of 31 meV but at a higher dose rate facilitating a dose range of 41 to 408 mGy. This resulted in the irradiation of human blood samples with thermal neutrons of approximately half the average energy of those in our prior study [19]. Part 1 of the results section addresses data for the DCA, micronucleus assay, and microdosimetry. Part 2 addresses a head-to-head comparison of biological findings for the present 31 meV and prior 64 meV thermal neutron spectra.

## Materials and methods

### Blood donation

The blood samples, drawn into vacuum-evacuated sodium citrate Vacutainers (BD, Franklin Lakes, NJ, USA), were from volunteer adult donors between the ages of 30 to 49 years with no self-reported underlying health conditions. All blood donors indicated feeling healthy on the day of the blood draw, were non-smokers, had no known underlying health conditions, and had no history of radiotherapy or chemotherapy treatment. Two male and three female donors participated in the DCA study (denoted as donors A–E), and two males and one female participated in the micronucleus study (donors B, C, E). Following venipuncture, 4.2 ml of whole blood was aseptically aliquoted into custom-fabricated quartz test tubes, and then immediately transported to the irradiation facility. Veritas Independent Review Board (Montreal, QC, Canada) granted ethical approval.

### Neutron irradiation

**Figure 1:**
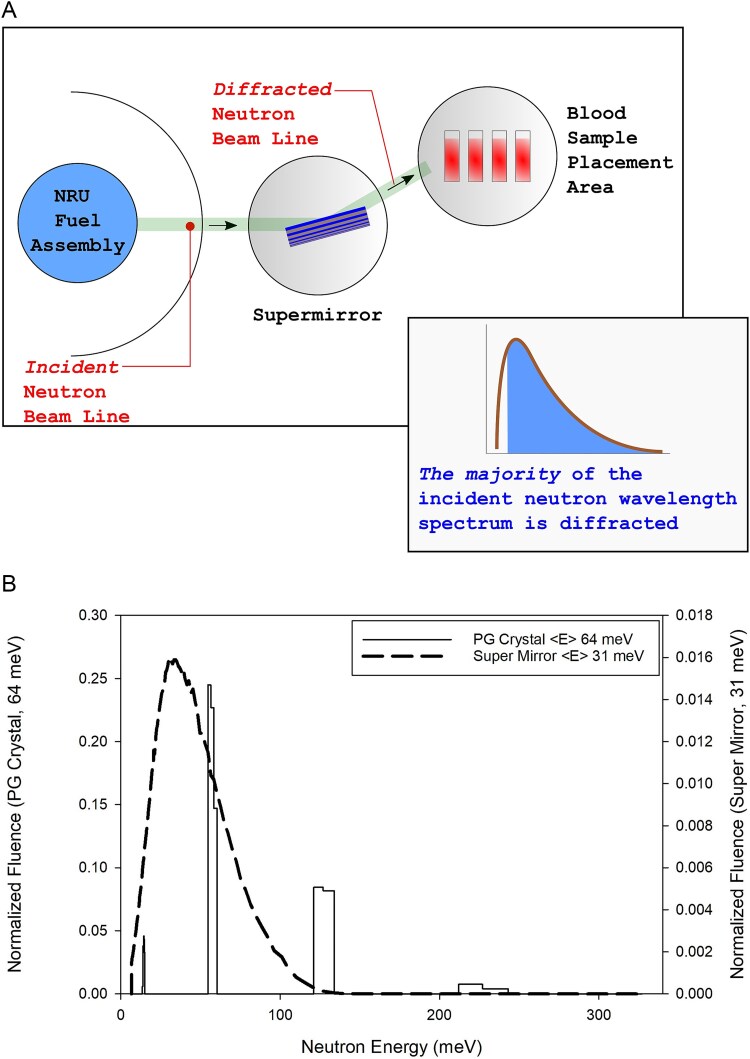
(A) Illustration of the NRU E3 beam line including super mirror and blood tubes. (B) Comparison of thermal neutron spectra for PG crystal (multi-modal solid line, average energy 64 meV) and super mirror (continuous dashed line, average energy 31 meV). Data modified from Ali *et al*. [11].

The 31 meV thermal neutron irradiations were completed using the Canadian Neutron Beam Centre’s E3 spectrometer located in CNL’s NRU reactor facility. Ali *et al*. [11] previously provided a comprehensive description of the beam-line configuration and physical modeling of the irradiation set-up illustrated in Fig. 1A. Briefly, a super mirror, composed of multiple layers of nickel and titanium, within the E3 beam-line selected for a 31 meV thermal neutron spectra, as illustrated by the dashed-line neutron energy spectrum in Fig. 1B. Extraneous photon dose from the beam-line was found to be negligible. Blood was modeled according to the guidelines set out in McConn Jr. *et al*. [9]. The modeling included the following mass fractions of isotopes: 10.19% ^1^H, 2.96% ^14^N, 75.94% ^16^O, and 10.00% C (mainly ^12^C). The total absorbed dose to the blood sample per unit neutron fluence was 0.331 pGy cm^2^ n^−1^ [10].

Similar to our prior irradiation [19], the quartz test tubes were affixed to a gantry in front of the beam port and rotated at 60 rpm to ensure a homogenous sample exposure. Blood samples were maintained at room temperature during irradiation. The neutron fluence rate varied with time due to reactor power changes and was calculated individually for each sample. The average thermal neutron fluence rate throughout the irradiation campaign was 1.60 × 10^8^ ± 0.01 × 10^8^ n cm^−2^ s^−1^, resulting in an average sample absorbed dose rate of 191 ± 2 mGy h^−1^. The fluence rate was verified using gold foil activation analysis. Total absorbed doses ranged from 41 to 408 mGy. The ^14^N(n,p)^14^C reaction contributed 71% of the total absorbed dose in this test system [11]. The remaining dose was a result of tertiary electrons that were liberated by secondary gamma rays that originated from the quartz test tube wall and blood volume. Whole blood cell cultures were established 18 h post-irradiation following room temperature incubation. This time interval ensured sufficient radionuclide decay prior to sample handling.

### Biological assays

#### Dicentric chromosome assay

**Figure 2:**
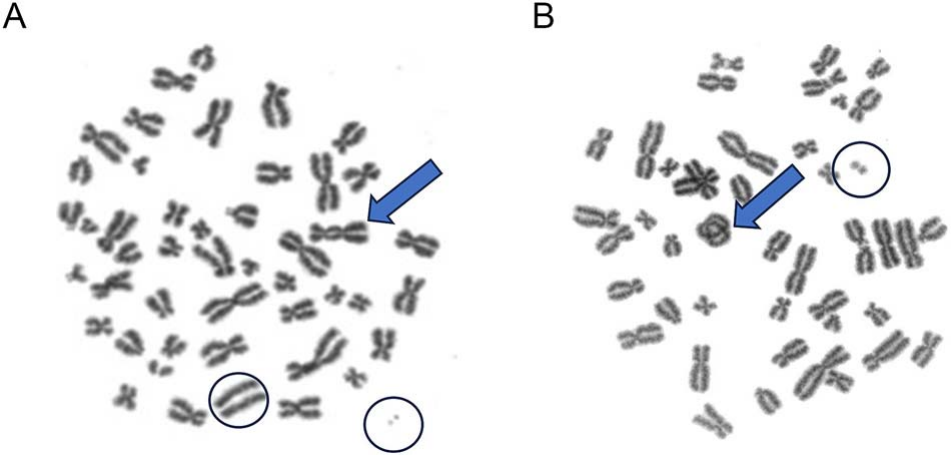
Examples of (A) dicentric and (B) ring chromosome aberrations (arrows). Both types of aberrations are accompanied by one or more associated acentric fragments (circled).

Duplicate whole blood cell cultures were established in Nunc T-25 flasks (Thermo Fisher Scientific Inc., Waltham, MA, USA) according to the International Atomic Energy Agency (IAEA) cytogenetic dosimetry guidelines and as previously described by Paterson *et al*. [20]. Briefly, cell cultures were incubated for 48 h, with colcemid (Thermo Fisher Scientific Inc., Waltham, MA, USA) present for the final 4 h. Samples were then harvested, placed on slides, and stained for microscopy. Slides were blinded and imaged under 630× magnification using the Metafer automated microscopy platform (Metasystems Group Inc., Newton, MA, USA). Images of complete metaphase spreads containing 46 centromeres that were arrested during first cell division were scored manually according to criteria described by Paterson *et al*. [20]. Dicentric chromosomes and ring chromosomes (Fig. 2A and Fig. 2B) were both included in the aberration tally.

#### Cytokinesis-block micronucleus assay

**Figure 3:**
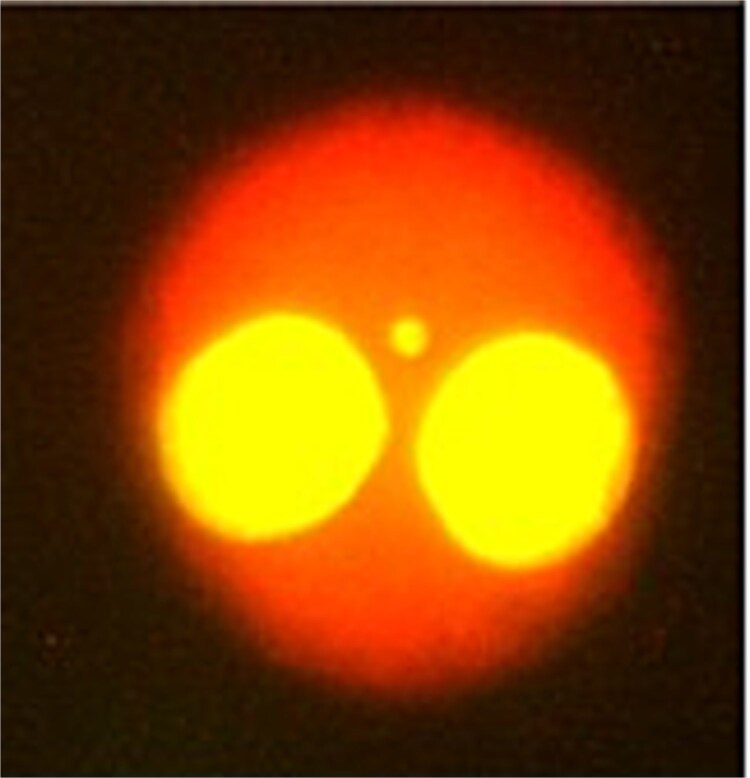
Example of a binucleated cell containing one micronuclei.

Whole blood cell cultures were established in Nunc T-25 flasks (Thermo Fisher Scientific Inc., Waltham, MA, USA) according to a modified procedure of Fenech *et al.* [21], previously described by McNamee *et al*. [22]. Briefly, cell cultures were incubated for 72 h, with cytochalasin B (Millipore Sigma, Burlington, MA, USA) added for the final 28 h. Samples were then harvested, placed on slides, and stained with acridine orange to facilitate fluorescence microscopy. Bi-nucleated cells on blinded slides, like those in Fig. 3, were manually scored under 400× magnification according to criteria provided by Fenech *et al*. [21].

### Microdosimetry

Ali *et al*. [11] demonstrated the use of a tissue equivalent proportional counter (TEPC) to quantify the quality factor of both the PG crystal and the super mirror thermal neutron beams. The implicit assumption in this approach is that the constituents of the TEPC, namely propane-based tissue-equivalent gas and Shonka A150 tissue-equivalent plastic, are similar to blood from a radiation energy deposition standpoint and that the TEPC is a microcosm of a blood volume. For the current study, additional TEPC response simulations were carried out using the Geant4 Monte Carlo system, an open source code, developed at the CERN laboratories. This quantified the quality factor changes with exposure to the primary, uncollided neutron field from the NRU reactor and with exposure to the collided neutron field and collided secondary gamma-ray field that traversed an individual blood volume.

The TEPC response simulations, described subsequently, were carried out using Geant4 version 4.10.06 with patch 01. The physics list QGSP_BIC_HP utilizes high-precision neutron cross-section files for energies between 0–20 MeV and the capability of the code to model the emission and transport of the secondary proton and residual ^14^C nucleus, emitted from the ^14^N(n,p)^14^C thermal neutron capture reaction. Standard electromagnetic physics option 3 was used. The physics lists for electromagnetic and hadronic processes were separately implemented. The detector response was modeled to the flux of neutrons and gammas in the energy range given by Ali *et al.* [11]. During the simulation, the particle range cut was reduced to 1 nm within the 0.127 mm thin plastic shell of the TEPC detector.

Two sets of TEPC response simulations were performed. The first set of simulations were completed using Geant4 code and evaluated the primary, uncollided thermal neutron energy spectrum for the PG crystal and super mirror beam-line inserts. For these uncollided spectra, the neutron source in these TEPC response simulations was represented as a planar mono-directional source whereby neutrons emanate from the plane through an angle of 0° with respect to the plane’s normal axis.

Using the MCNP model already developed for Ali *et al.* (2018) [11], the second set of TEPC simulations (carried out using the Monte Carlo N-Particle (MCNP) version 6.2 code [23]) first evaluated the collided neutron energy spectrum and the collided secondary gamma ray energy spectrum that traversed an individual blood volume when the PG crystal or super mirror beam line insert was used. Here, the primary, uncollided incident thermal neutron energy spectrum passed through the blood and the surrounding tube and underwent elastic scatter and absorption reactions, the latter of which may be (n,γ) capture reactions.

The energy-integrated collided neutron and collided secondary gamma-ray fluence (normalized per source neutron) traversing the blood volume was noted and are represented as *Φ*_n_ and *Φ*_γ_, respectively. The subsequent TEPC simulations in Geant4 had the TEPC separately irradiated by the collided neutron energy spectrum and by the collided secondary gamma-ray energy spectrum. Here, the TEPC was surrounded by a slightly larger sphere whereby neutrons were emitted from the inner surface toward the TEPC through a variety of angles, with the previously mentioned collided neutron energy distribution. The same simulation was repeated for collided secondary gamma rays.

This yielded a pulse height spectrum for events within the TEPC sensitive gas volume. Let *C*_*i*,n_ and *C*_*i*,γ_ denote the counts per source neutron and gamma ray, respectively, in the *i*^th^ energy deposition bin of their pulse height spectra. Then the counts in the *i*^th^ bin normalized to the strength of the collided neutron field and collided secondary gamma-ray field are calculated as (*Φ*_γ_/(*Φ*_n_ + *Φ*_γ_)) × *C*_*i*,γ_ + (*Φ*_n_/(*Φ*_n_ + *Φ*_γ_)) × *C*_*i*,n_. Using this unified pulse height spectrum, the following microdosimetric metrics were obtained: frequency mean lineal energy ($\bar{y} $_F_), dose mean lineal energy ($\bar{y} $_D_), quality factor (*Q*), and dose distribution.

If a blood volume is assumed to be composed of numerous spherical 1 μm individual microscopic sub-volumes, the “hit” probability distribution (*ν*) can be calculated [24]. This parameter quantifies both the hits to the sub-volume and the fraction of all microscopic sub-volumes within the overall blood volume experiencing a certain number of hits. Let *z-*_F_ represent the most frequent absorbed dose delivered by an individual hit and *D* the absorbed dose delivered to the overall blood volume and to each constituent microscopic sub-volume (assuming spatially uniform dose delivery throughout the blood volume). Then the mean number of hits in an individual microscopic sub-volume is given by *N* = *D*/*z-*_F_. The previously mentioned probability for *ν* hits to occur is given by *p*(*ν*) = *N^ν^*e^−*N*^/*ν*!.

### Statistics

Statistical data analysis was completed in accordance with the IAEA cytogenetic dosimetry guidelines [13]. The DCA and micronucleus assay aberration distributions were tested for agreement with the Poisson distribution. A dispersion index (variance, *σ*^2^/mean, *y*) of unity indicated compliance with the Poisson distribution. Dispersion indices beyond unity were further examined using the *u*-test, where a statistic above 1.96 indicated non-Poisson over-dispersion at the 5% significance level [13, 25].

Dose-response curve fitting was achieved using the Dose Estimate software (version 5.2) [26]. Regression is presented in the form *A* = *c* + *αD* + *βD*^2^ (linear-quadratic) or *A* = *c* + *αD* (linear), where *A* is the frequency of aberrations at a given dose point, *c* is the background frequency of aberrations, *α* is the linear dose-response coefficient, *β* is the quadratic dose-response coefficient, and *D* is the absorbed dose in Gy. Dose-response curve goodness of fit was tested using the chi-squared test (*χ*^2^). Dose-response equation coefficients and RBE values were evaluated using the *z*-test. A *z*-test coefficient with a *P*-value <0.05 was considered statistically significant. Errors were reported as either standard deviation (SD) or standard error of the mean (SE).

### Relative biological effectiveness calculation

RBE was calculated using two methods: the maximum RBE at minimal doses, or RBE_M_, method and the dose-specific RBE method. Dose-specific RBE values were calculated as the ratio of gamma and thermal neutron doses required to produce the same biological effect. RBE_M_ was calculated as the quotient of the thermal neutron dose-response curve regression equation *α* coefficient and the photon reference radiation dose-response curve regression equation *α* coefficient, as described in the IAEA cytogenetic dosimetry guidelines [13].

## Results

### Part 1: 31 meV thermal neutron exposures

#### Dicentric chromosome assay

The distribution of dicentric and ring chromosomes in human lymphocytes following whole blood exposure to a thermal neutron spectrum with an average energy of 31 meV (for brevity, referred to as only 31 meV) is presented in Table 2. A total of 12643 cells, limited by sample size, were pooled from five donors and evaluated across six dose points between 0 and 408 mGy. The background level of aberrations was 0.001 aberrations per cell, and the maximum aberration yield was 0.204 aberrations per cell for the highest dose of 408 mGy. The majority of cells exposed had no aberrations, and ~17% of cells at the highest dose point contained between one and four aberrations. All five irradiated dose points demonstrated non-Poisson over-dispersion, evidenced by *u*-test statistic values beyond 1.96. This is a result of a higher-than-expected proportion of cells having multiple aberrations beyond what is predicted by the Poisson distribution.

**Table 2:** DCA aberration distribution in blood lymphocytes from five donors following exposure to thermal neutrons with an average energy of 31 meV. Values of the *u*-test statistic demonstrating non-Poisson over-dispersion are highlighted in bold.

Total dose (mGy)	(n,p) dose (mGy)	(n,γ) dose (mGy)	Cells scored	Aberration type			Dic + Ring (± SD)^*^	Cellular distribution of aberrations					Aberrations per cell			Disp. index (*σ*^2^/*y*)	*u*- test	Dose-specific RBE
				Dic.	Ring	Ace.		0	1	2	3	4	Total	(n.p) only	(n,γ) only			
0	0	0	2326	2	0	23	2 ± 1	2324	2	0	0	0	0.001	0.001	0.000	1.00	−0.02	–
41	29	12	988	17	3	43	20 ± 5	972	13	2	1	0	0.020	0.017	0.003	1.48	**10.97**	6
102	72	30	2217	88	7	172	95 ± 10	2134	71	12	0	0	0.043	0.035	0.008	1.21	**7.04**	4
204	144	60	2118	168	22	314	190 ± 14	1946	155	16	1	0	0.090	0.073	0.017	1.11	**3.61**	4
306	216	90	2607	340	29	591	369 ± 19	2287	278	35	7	0	0.142	0.113	0.027	1.16	**5.87**	3
408	288	120	2387	439	49	766	488 ± 22	1984	327	68	7	1	0.204	0.166	0.038	1.19	**6.41**	3

Dic., dicentric chromosome; Ace., acentric chromosome; SD, standard deviation; Disp., dispersion; ^*^assuming Poisson distribution.

**Figure 4:**
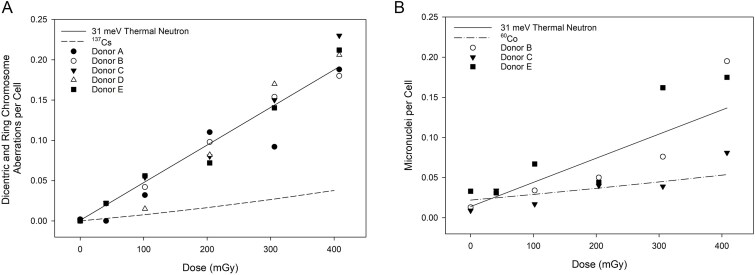
(A) Frequency of dicentric and ring chromosome aberrations per cell following exposure to thermal neutrons with an average energy of 31 meV compared to the ^137^Cs dose-response curve (dashed line) by Flegal *et al*. [27]. (B) Frequency of micronuclei per cell following exposure to thermal neutrons with an average energy of 31 meV compared to the ^60^Co dose-response curve (dashed-dot line) by McNamee *et al*. [22]. For both (a) and (b), the solid line represents the 31 meV dose-response derived from the pooled data.

Fig. 4A illustrates the 31 meV thermal neutron linear dose-response curve derived for the pooled DCA data, compared to the same dose range of the linear-quadratic ^137^Cs reference radiation dose-response curve. This ^137^Cs dose-response curve was previously completed utilizing a dose rate of 801 mGy min^−1^ [27]. The data points on Fig. 4A represent the five independent donors. Individual DCA donor data has been included in Supplement S2. The SE of the mean aberrations per cell across the five donors was low due to the tight grouping of donor data.

The regression coefficients and SE values, as well as the results of the *χ*^2^- and *z*-tests and the *R*^2^ values generated from the pooled DCA data are presented in Table 3. Both linear and linear-quadratic regression fits were evaluated. Whereas the linear *α* regression coefficients (column 8, Table 3) both tested as significant for both regression fits, the quadratic *β* regression coefficient (column 9, Table 3) did not adequately fit the data. This indicated a clear preference for the linear regression fit. The *χ*^2^-test *P*-values given in column 7 of Table 3 indicate that the regression fit was not statistically different from the observed DCA data points, further confirming a good linear regression fit. Individual DCA donor statistics are presented in Supplement S3.

**Table 3:** Dose-response regression values and statistical testing results for 31 meV thermal neutrons for the DCA and the micronucleus assay.

Dicentric chromosome assay									
Radiation	Regression	(*A* = *c* + *αD* + *βD*^2^)	***α*** [±SE] (Gy^−1^)	***β*** [±SE] (Gy^−2^)	*c* [±SE]	*χ* ^2^-test sig.	*α z*-test sig.	*β z*-test sig.	Pearson’s *R*^2^ value
31 meV neutron	Linear	*A* = 0.0008 + 0.467*D*	0.467 ± 0.015	–	0.0008 ± 0.0012	0.668	< 0.001	–	1.0
	Linear-quadratic	*A* = 0.0009 + 0.387*D* + 0.259*D*^2^	0.387 ± 0.042	0.259 ± 0.131	0.0009 ± 0.0006	0.865	0.003	0.14	1.0
**Micronucleus assay**									
**Radiation**	**Regression**	**(*A* = *c* + *αD* + *βD*^2^)**	***α*** **[±SE] (Gy^−1^)**	***β*** **[±SE] (Gy^−2^)**	***c*** **[±SE]**	***χ* ^2^-test** **sig.**	***α z*-test sig.**	***β z*-test sig.**	**Pearson’s *R*^2^ value**
31 meV neutron	Linear	*A* = 0.014 + 0.298*D*	0.298 ± 0.043	–	0.014 ± 0.011	–	0.002	–	0.96
	Linear-quadratic	*A* = 0.018 + 0.111*D* -0.555*D*^2^	0.111 ± 0.123	0.555 ± 0.361	0.018 ± 0.006	–	0.435	0.222	0.98

*A*, aberrations per cell; *α, β, c*, regression coefficients; *D*, dose (Gy); *R*^2^, coefficient of determination; Sig., significance.

#### Cytokinesis-block micronucleus assay

The distribution of micronuclei in bi-nucleated human lymphocytes following exposure to 31 meV thermal neutrons is presented in Table 4. A total of 22007 bi-nucleated cells from three donors were scored across six dose points between 0 and 408 mGy. The background level of aberrations was 0.015 aberrations per cell, and the maximum aberration yield was 0.157 aberrations per cell for the highest dose point of 408 mGy. The majority of cells exposed had no aberrations. Approximately 13% of cells at the highest dose point contained between one and three micronuclei. All dose points demonstrated *u*-test statistic values beyond 1.96, indicating non-Poisson over-dispersion. This is a result of a higher-than-expected proportion of cells having multiple aberrations. This is typical for the micronucleus assay due to the way the damage is enumerated, even in low-LET exposure scenarios [13].

**Table 4:** Micronuclei distribution in blood lymphocytes from three donors following exposure to thermal neutrons with an average energy of 31 meV. Values of the *u*-test statistic demonstrating non-Poisson over-dispersion are highlighted in bold.

Total dose (mGy)	BNCs scored	Total MN (± SD)^*^	Cellular distribution of MN				Total MN Per Cell	Disp. index (*σ*^2^/*y*)	*u*-test	Dose-specific RBE
			0	1	2	3				
0	5634	82 ± 9	5556	74	4	0	0.015	1.08	**4.44**	–
41	3000	95 ± 10	2913	80	6	1	0.032	1.16	**6.16**	6
102	2826	115 ± 11	2722	94	9	1	0.041	1.17	**6.36**	3
204	3537	161 ± 13	3389	136	11	1	0.046	1.13	**5.43**	2
306	3807	423 ± 21	3448	300	54	5	0.111	1.22	**9.41**	3
408	3203	504 ± 23	2796	315	87	5	0.157	1.25	**9.92**	3

BNCs, bi-nucleated cells, MN, micronuclei, Disp., dispersion; SD, standard deviation; ^*^assuming Poisson distribution.

Fig. 4B illustrates the derived 31 meV thermal neutron linear micronucleus dose-response curve for the pooled data compared to the same region of the previously completed linear-quadratic ^60^Co reference radiation dose-response curve. For the ^60^Co dose-response curve, the 0.1, 0.25 and 0.50 Gy exposures were at a dose rate of 186 mGy min^−1^, whereas the 1, 2, 3 and 4 Gy exposures were at a dose rate of 8832 mGy min^−1^. The Fig. 4B data points represent three independent donors. Individual micronucleus donor data has been included in Supplement S4. The SE of the mean for most dose points (mean: number of aberrations per cell) was larger for the micronucleus assay than for the DCA assay, owing to the micronucleus assay’s greater inter-individual variability [28].

The linear regression coefficients and SE values, as well as the results of the *χ*^2^- and *z*-tests and the *R*^2^ values, are presented in Table 3. As with the DCA, both linear and linear-quadratic regression fits were evaluated. The linear regression *α* coefficients tested significant, whereas the quadratic *β* regression coefficient (column 9, Table 3) did not adequately fit the data, indicating a clear preference for the linear regression fit. Individual micronucleus donor statistics are presented in Supplement S3.

#### Relative biological effectiveness

A DCA RBE_M_ of 6.7 ± 0.9 was evaluated in reference to ^137^Cs radiation [27] (^137^Cs *α*: 0.070 ± 0.0088). An inverse dose effect was observed for dose-specific RBE with values that decreased with increasing dose from 6 at 41 to 3 at 408 mGy (Table 2).

An RBE_M_ of 4.4 ± 0.7 was evaluated for the micronucleus assay in reference to ^60^Co radiation [22] (^60^Co *α*: 0.068 ± 0.006) and an inverse dose effect was observed for dose-specific RBE with values that decreased with increasing dose from 6 at 41 mGy to 2 at 204 mGy (Table 4).

In an attempt to remove the biological effect caused by the (n,γ) capture reactions in the RBE_M_ value, a method described by Schmid *et al*. [18] was employed to evaluate the DCA RBE_M_ for only the effect of the (n,p) capture reaction (RBE_M(n,p)_). This resulted in a RBE_M(n,p)_ of 7.6 ± 1.0 and required the assumption that the photon spectrum produced by the thermal neutron (n,γ) capture reactions produced the same biological consequence as the spectrum used in ^137^Cs (DCA) gamma irradiations. The RBE_M(n,p)_ of 7.6 ± 1.0 differs significantly from the 64 meV RBE_M(n,p)_ of 15.5 ± 2.2 [19] and from the quality factor (*Q*) of ~19 that were derived for the ^14^N(n,p)^14^C neutron capture reaction alone [11].

### Part 2: Current 31 meV data in comparison with prior 64 meV data

One benefit of this study is the ability to compare RBE values for two different thermal neutron energy spectra (31 and 64 meV [19]) when most other experimental variables were kept constant (Table 5).

**Table 5:** Parameter comparison for the 64 meV and 31 meV thermal neutron exposures. The exposure environment, cell culture, and microscopy conditions were identical and are described in the Materials and methods section.

Beam-line insert	PG crystal	Super mirror
Mean energy	64 meV	31 meV
Spectrum	Multi-modal	Continuous
Average dose rate	22 mGy h^−1^	191 mGy h^−1^
Dose range	6–85 mGy	41–408 mGy
Mean fluence rate	2.25 × 10^7^ n cm^−2^ s^−1^	1.60 × 10^8^ n cm^−2^ s^−1^
DCA dose-response slope	0.789 ± 0.045 Gy^−1^	0.467 ± 0.015 Gy^−1^
Avg. non-Poisson *u*-test statistic (DCA, micronucleus assay)^a^	7.17, 22.40	6.78, 7.46
RBE (DCA, micronucleus assay)	11.3 ± 1.6, 9.0 ± 1.1	6.7 ± 0.9, 4.4 ± 0.7
Quality factor	14.98	15.02

^a^Not including 0 Gy dose point.

The 31 meV irradiations produced significantly different DCA and micronucleus results compared to prior 64 meV irradiations [19]. The linear slope coefficient of the linear-quadratic DCA dose-response curve for the 31 meV exposures (0.467 ± 0.015 Gy^−1^) was significantly depressed compared to the 64 meV dose-response curve slope (0.789 ± 0.045 Gy^−1^, *z* = 6.79, *P* < 0.01). The same trend was found for the micronucleus data sets, with significantly different linear slope coefficients (*z* = 4.70, *P* < 0.01) for the 31 meV (0.298 ± 0.043 Gy^−1^, Table 3) and 64 meV (0.615 ± 0.052 Gy^−1^) exposures.

For the 31 meV and 64 meV thermal neutron irradiations, all five irradiated 31 meV DCA dose points (Table 2) and five of the seven irradiated 64 meV DCA dose points [19] demonstrated non-Poisson over-dispersion, as evaluated by the u-test. The magnitude of the over-dispersion, and hence aberration clustering, was similar for the 64 meV DCA samples (range: 6.03 to 8.34, mean: 7.17, SD: 1.02), and the 31 meV samples (range: 3.61 to 10.97, mean: 6.78, SD: 2.67). However, the micronucleus data showed divergent u-test values for the 64 meV irradiations (range: 19.89 to 23.53, mean: 22.40, SD: 1.71) and the 31 meV irradiations (range: 5.43 to 9.92, mean: 7.46, SD: 2.06).

Differences between the 64 meV and 31 meV thermal neutron spectra also emerged when RBE was evaluated. For the 31 meV irradiations, RBE_M_ values of 6.7 ± 0.9 and 4.4 ± 0.7 were found for the DCA and the micronucleus assay, respectively. This was approximately two-fold lower than our previously reported 64 meV DCA RBE_M_ value of 11.3 ± 1.6 (*z* = 2.51, *P* = 0.01) and 64 meV micronucleus RBE_M_ of 9.0 ± 1.1 (*z* = 3.53, *P* < 0.01).

**Figure 5:**
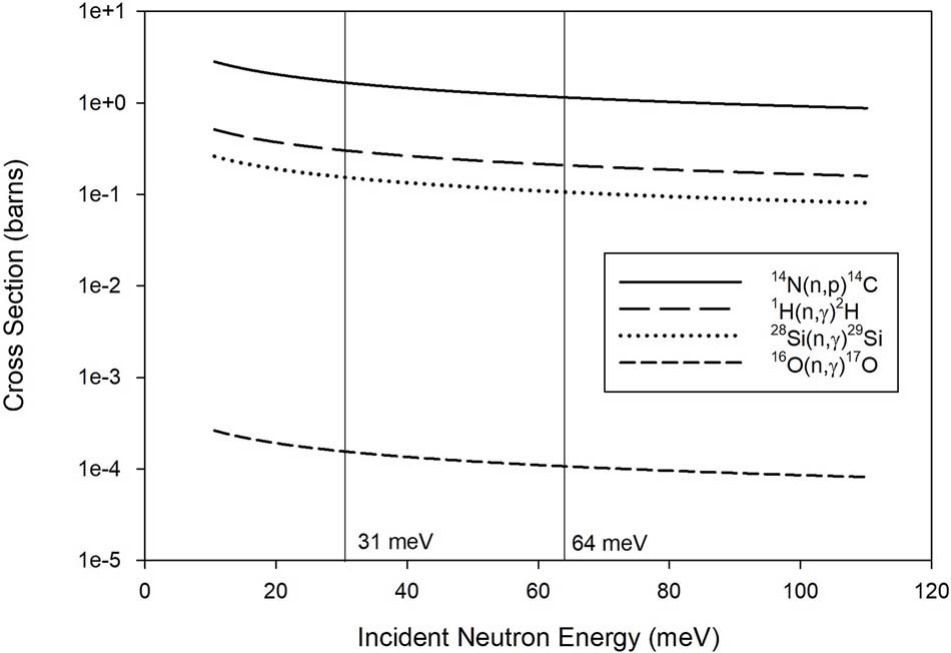
^1^H, ^14^N, ^16^O, and ^28^Si neutron capture cross-section variation across the thermal energy range. Data from IAEA ENDF B-VII.0 library [29]. Carbon is not shown as the dose contribution from the (n,γ) capture reactions is not significant to the test system described here.

In the test system presented here, dose is primarily delivered through four neutron capture reactions: ^1^H(n,γ)^2^H, ^14^N(n,p)^14^C, ^16^O(n,γ)^17^O, and ^28^Si(n,γ)^29^Si (Equation 1). The ^1^H and ^14^N isotopes present in the blood are responsible for the bulk of the absorbed dose. Neutron capture cross-sections vary considerably across the thermal energy range (Fig. 5). For example, there is an approximately two-fold greater ^14^N neutron capture cross-section for 20 meV neutrons compared to 100 meV neutrons. The lower-energy portion of the 31 meV super mirror spectrum coincides with increased cross-section values for ^1^H, ^14^N, ^16^O, and ^28^Si, in comparison to our prior 64 meV thermal neutron irradiations, as illustrated in Fig. 5. When the 31 meV super mirror spectrum irradiates the tube containing blood, compared to the 64 meV PG crystal spectrum, two effects are noted. First, there are more ^14^N(n,p)^14^C capture reactions, which leads to a higher absorbed dose delivered to the blood (Table 1 of Ali *et al*. [11]) for the 31 meV neutron spectra (absorbed dose per unit neutron fluence of 0.234 pGy cm^2^ n^−1^) compared to the 64 meV PG spectra (absorbed dose per unit neutron fluence of 0.194 pGy cm^2^ n^−1^). Second, more (n,γ) reactions occur in the blood and tube, which leads to more low-LET events contributing to the absorbed dose delivered to the blood. This is illustrated in the tertiary electron absorbed dose per unit neutron fluence of 0.097 pGy cm^2^ n^−1^ for the 31 meV super mirror spectrum compared to the 0.080 pGy cm^2^ n^−1^ for the 64 meV PG spectrum (Table 1 of Ali *et al*. [11]). It is proposed that the higher frequency of low-LET (n,γ) events resulting from the 31 meV super mirror spectrum weighed down the overall RBE for dose delivery from all particle types, resulting in a lower RBE for the 31 meV super mirror spectrum compared to the 64 meV PG crystal spectrum. Overall, the ^14^N(n,p)^14^C dose contribution relative to the (n,γ) dose contribution is very similar for the 31 meV and 64 meV irradiations, with the ^14^N(n,p)^14^C contributing ~71% of the absorbed dose and the (n,γ) reactions contributing ~29%.

#### Microdosimetry

**Figure 6:**
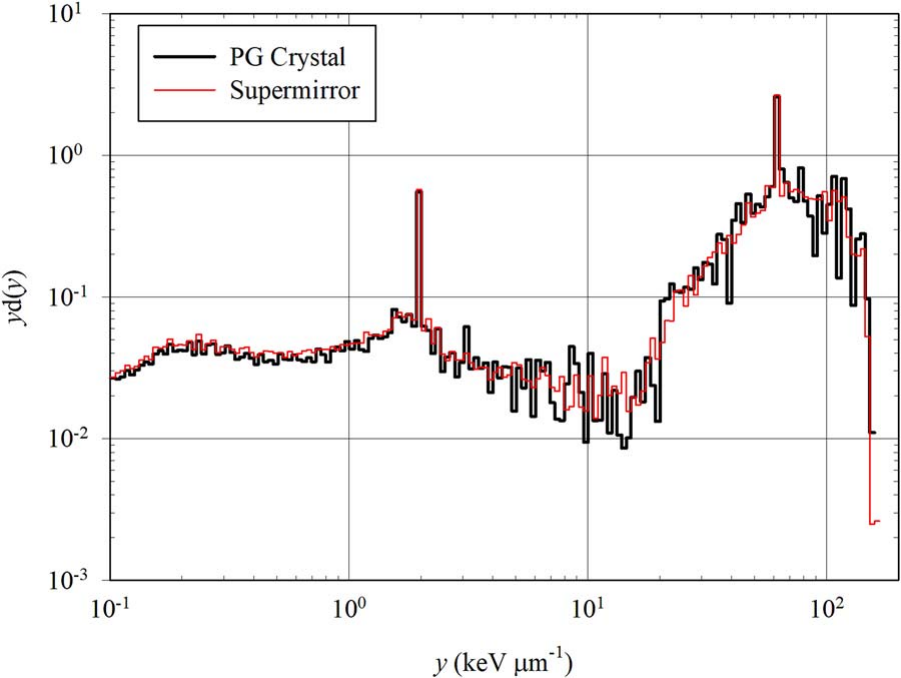
TEPC dose distributions accounting for collided neutrons and collided secondary gamma rays for PG crystal and super mirror use.

The neutron fluence traversing the blood volume was similar for the super mirror (0.177 neutrons cm^−2^ source n^−1^) and the PG crystal beam-line insert (0.178 neutrons cm^−2^ source n^−1^). The secondary gamma-ray fluence traversing the blood volume was ~20% higher when using the super mirror (0.00265 gamma rays cm^−2^ source n^−1^) than with the PG crystal beam-line insert (0.00220 gamma rays cm^−2^ source n^−1^). Table 6 tabulates the total neutron fluence passing through the blood (normalized per source neutron used in the initial MCNP simulations) and the total secondary gamma-ray fluence passing through the blood (normalized per source neutron used in the initial MCNP simulations) for each scattering device. The data in Table 6 indicate that regardless of which scattering device was used, accounting for the secondary gamma-ray field noticeably weighs down each metric toward lower lineal energies (for frequency mean lineal energy, $\bar{\text{y}} $_F_, and dose-mean lineal energy, $\bar{\text{y}} $_D_) and toward lower quality factors (since gamma rays have a quality factor of one). When comparing the metrics, accounting for neutrons and secondary gamma rays, the quality factor for the super mirror is only ~0.3% lower than that for the PG crystal; this may be due to a stronger secondary gamma-ray field that passed through the blood when the super mirror was used. Fig. 6 displays the TEPC dose distributions accounting for collided neutrons plus collided secondary gamma rays when the PG crystal and super mirror are used. Gamma rays induce detection events with lineal energies between 0.10 and 10 keV μm^−1^, thermal neutrons induce detection events between ~10 and 100 keV μm^−1^, and fast neutrons can induce detection events between 10 and 1000 keV μm^−1^.

**Table 6:** Microdosimetric metrics for blood irradiations accounting for the neutron and secondary gamma fields.

Metric	PG crystal *Uncollided neutrons*	PG crystal *Collided neutrons + secondary gamma rays*	Super mirror *Uncollided neutrons*	Super mirror *Collided neutrons + secondary gamma rays*
* $\bar{{y}} $ *_F_ (keV μm^−1^)^a^	6.44	2.53	6.74	2.35
* $\bar{{y}} $ *_D_ (keV μm^−1^)^b^	66.17	55.81	68.60	52.29
$\bar{\text{Q}} $ ^c^	18.42	14.98	18.98	15.02
$\bar{z} $ _F_ (mGy)^d^	1313.76	516.12	1374.96	479.40

^a^frequency mean lineal energy; ^b^dose-mean lineal energy; ^c^quality factor; ^d^frequency mean specific energy.

Given that the TEPC is capable of measuring a wide range of lineal energies, the Fig. 6 dose distribution seeks to sort these lineal energies into many bins. For any specific bin, *y*_D_(*y*) is evaluated and is equal to the fraction, normalized to the bin width on a logarithmic scale, of the total dose measured by the TEPC due to detection events with lineal energies that fall within the bin in question. It can be seen from the dose distribution that thermal neutrons deliver the majority of the dose to the TEPC.

Table 7 tabulates the hit probability distribution for the PG crystal and super mirror and for either neutron and secondary gamma-ray dose delivery or only neutron dose delivery. The probability of one or more hits to the microscopic sub-volume increases with increasing dose. Higher absorbed doses were delivered to the overall blood volume when using the super mirror, compared to the PG crystal. As a result, the value of *N*, the probability of one hit, and the probability of greater than one hit are elevated for the super mirror compared to the same metrics for the PG crystal, suggesting a greater spatial density of charged particle tracks and therefore hits throughout the overall blood volume as a result of super mirror use.

**Table 7:** Microdosimetric hit distribution for the PG crystal and super mirror.

PG Crystal									
Absorbed dose delivery from neutrons and secondary gamma rays					Absorbed dose delivery from neutrons only				
Total absorbed dose (mGy)	*N*	*p*(0)	*p*(1)	1 − *p*(0) − *p*(1)	(n,p) absorbed dose (mGy)	*N*	*p*(0)	*p*(1)	1 − *p*(0) − *p*(1)
6.0	0.012	0.988	0.011	0.000	4.20	0.003	0.997	0.003	0.000
12.0	0.023	0.977	0.023	0.000	8.50	0.006	0.994	0.006	0.000
21.0	0.041	0.960	0.039	0.001	14.90	0.011	0.989	0.011	0.000
23.9	0.046	0.955	0.044	0.001	16.90	0.013	0.987	0.013	0.000
42.2	0.082	0.921	0.075	0.003	30.20	0.023	0.977	0.022	0.000
61.9	0.120	0.887	0.106	0.007	43.80	0.033	0.967	0.032	0.001
82.1	0.159	0.853	0.136	0.011	58.10	0.044	0.957	0.042	0.001
**Super mirror**									
**Absorbed dose delivery from neutrons and secondary gamma rays**					**Absorbed dose delivery from neutrons**				
**Total absorbed dose (mGy)**	***N***	***p*(0)**	***p* (1)**	**1 − *p*(0) − *p* (1)**	**(n,p) absorbed dose (mGy)**	***N***	***p*(0)**	***p* (1)**	**1 − *p*(0) − *p* (1)**
41	0.086	0.918	0.079	0.003	29	0.021	0.979	0.021	0.000
102	0.213	0.808	0.172	0.020	72	0.052	0.949	0.050	0.001
204	0.426	0.653	0.278	0.069	144	0.105	0.901	0.094	0.005
306	0.638	0.528	0.337	0.135	216	0.157	0.855	0.134	0.011
408	0.851	0.427	0.363	0.210	288	0.209	0.811	0.170	0.019

*N*, mean number of hits in an individual microscopic sub-volume of blood; *p*(0), probability of 0 hits; *p* (1), probability of one hit; 1 – *p*(0) – *p* (1), probability of greater than one hit.

The data in Table 7 illustrate that at the similar absorbed doses of 42 mGy (64 meV, PG crystal) and 41 mGy (31 meV, super mirror), all metrics for absorbed dose from combined thermal neutrons and secondary gamma rays, as well as from only neutrons, were nearly identical. This indicates that the doses delivered to the microscopic sub-volumes are similar for the two irradiation configurations.

## Discussion

**Figure 7:**
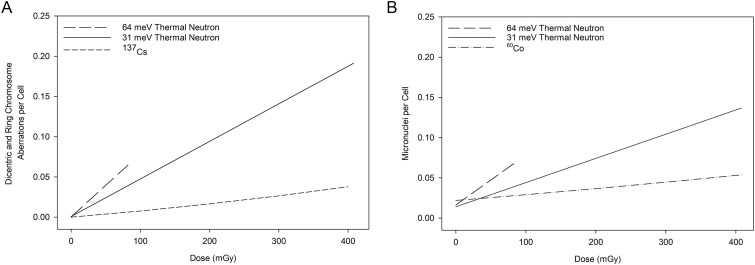
Significantly different dose-response curve slopes were obtained for the (A) DCA and (B) micronucleus assay following exposure to a thermal neutron spectrum with mean energies of 31 and 64 meV.

The reported thermal neutron RBE for the DCA varies greatly from 5 to 51 [15–19]. To investigate a possible source of the reported RBE variation, the present study and the prior 64 meV study were completed using similar cell culture, cell harvest, and microscopy protocols (Table 5). Furthermore, RBE was calculated using the same reference radiation dose-response curves. Only the dose rates and the thermal neutron energy spectra varied greatly. Yet, the thermal neutron dose-response curve slopes (Fig. 7A and Fig. 7B) and thus the RBE_M_ were approximately two-fold lower for the 31 meV than for the 64 meV thermal neutral spectra, for both the DCA and the micronucleus assay. An important question has come from this work: what is the reason for the difference between the 31 meV and 64 meV RBE? The first two subsections below outline our answer, and further subsections describe dose effects and the radiation protection implications of this work.

### Are the 31 and 64 meV relative biological effectiveness differences due to protocol inconsistencies?

The overall donor age range was similar for both studies: the 31 meV study utilized blood from donors ranging in age from 30 years to 49 years while the 64 meV study utilized blood from donors ranging in age from 29 years to 49 years. All donors regularly donated blood for the biological dosimetry program at CNL. It was desirable, but not possible, to obtain blood from identical donors for both the 31 meV and 64 meV studies with the exception of one donor. To date, no individual radiosensitivity or radioresistance has been noted in these donors during regular DCA scoring competency exercises (unpublished data). The 31 meV and the 64 meV studies were both completed before the severe acute respiratory syndrome coronavirus 2 (SARS-CoV-2) global pandemic. As such, the lymphocytes used in either the 31 or 64 meV study were not affected by downstream effects of the SARS-CoV-2 infection.

The protocols described in this publication for the DCA and the micronucleus assay are identical to those of our previous publication on 64 meV thermal neutrons, adhere to the IAEA cytogenetic dosimetry guidelines [13], and are utilized as part of the biodosimetry program at CNL [30]. For the DCA, dicentric and ring chromosome aberrations were included in the aberration tally, to match the published ^137^Cs gamma-ray reference radiation DCA dose-response curve [27]. Recent biological dosimetry inter-comparisons have demonstrated DCA scoring accuracy when both rings and dicentric chromosomes were considered [31]. Furthermore, the use of two assays provided verification of the RBE results, as the RBE values for the DCA and micronucleus assays were of similar magnitude within each treatment group but were significantly different across the treatment groups.

The microscopy was completed by a qualified individual who is part of the Canadian biodosimetry network and who participated in prior DCA and micronucleus gamma curve scoring [30]. The sole-scorer method eliminates inter-individual scoring differences and can assist with low-error regression evaluations, contributing to highly accurate RBE evaluations. This is demonstrated here where the SE accounts for 4% of the DCA linear regression coefficient (Table 3) and 14% of the RBE_M_ for our 31 meV thermal neutron spectra (Table 1). It is also demonstrated in our prior report on 64 meV thermal neutrons, where the SE similarly accounts for 6% of the linear regression coefficient and 14% of the DCA RBE_M_ [19].

RBE calculations for both the 31 meV and 64 meV thermal neutron samples utilized the same reference radiation dose-response curves (^137^Cs for the DCA [27] and ^60^Co for the micronucleus assay [22]), to avoid introducing another confounding factor. It has been noted for future research that DCA and micronucleus data should be compared to the same reference radiation, and steps are being taken in this regard. However, this does not affect the RBE conclusions drawn here between the 31 meV and 64 meV thermal neutron datasets.

Given the points outlined above, it is unlikely that the differences seen for the 31 meV and 64 meV thermal neutron samples were due to protocol or microscopy differences.

### Are the 31 and 64 meV relative biological effectiveness differences due to irradiation characteristics?

Interestingly, as shown in Table 5, the 64 meV DCA and micronucleus assay both demonstrated elevated and significantly different chromosomal damage, slope, and RBE values compared to the 31 meV results. Theoretical evaluations of high-LET radiations have demonstrated that increased RBE is a consequence of elevated complex clustered DNA damage [32], which is consistent with our micronucleus findings. Reported evaluations of two epithermal sources with a small fast-neutron component concluded that minor differences in the neutron energy spectrum may be responsible for significant differences in surviving cells fraction and RBE [33]. It is possible that the same conclusion holds true for the current RBE evaluation.

The 64 and 31 meV studies were completed at low dose rates of 22 and 191 mGy h^−1^, respectively. For the 64 meV study, irradiation times varied from 0.25 h for 6 mGy to 4 h for doses of ~82 mGy. For the 31 meV study, irradiation times varied from 0.2 h for 41 mGy to 2 h for 408 mGy. In both irradiation set-ups, the ^14^N(n,p)^14^C capture reaction is responsible for ~71% of the dose, whereas the (n,γ) capture reactions are responsible for ~29% of the dose. The proton and ^14^C recoil nucleus high-LET single-track DNA damage is less influenced by dose rate than low-LET photon traversals. However, the photon dose component could have been modified by the protracted irradiations whereby a photon lesion was repaired before a second lesion occurred within the same nucleus. It is not currently clear whether the neutron spectra differences or the dose rate differences have the strongest influence on the RBE value. Future investigation into this variation of RBE with thermal neutron energy would be beneficial.

### Is there a relative biological effectiveness dose-effect?

For the DCA and micronucleus assay evaluated here and in the 64 meV paper, a trend toward an increase in RBE with decreasing dose was observed. This RBE dose effect had been observed previously following higher-energy neutron irradiations [20, 34, 35] using the DCA, following cold neutron exposures using a survival assay (from an RBE of 4 at 0.311 Gy to an RBE of slightly <2 at 4.977 Gy) [36], and following exposure to thermal neutrons (using the Schmid *et al.* [18] data, an RBE of 4 at 0.375 Gy and an RBE of 2 at 1.875 Gy were estimated). This dose effect is not surprising as it is indicative of a convergence in the slopes, whereby the quadratic component of the reference radiation dose-response curve causes an approach toward the linear neutron dose-response curve under study as doses increase.

The data from the 64 meV and 31 meV experiments demonstrate that even at low to moderate doses (<500 mGy) of thermal neutrons, the RBE dose effects can still be detected. Furthermore, our data demonstrate that dose-specific RBE becomes nearly equal to RBE_M_ at the lowest doses. This is a noteworthy finding insofar as RBE_M_ is not often observed experimentally and thus direct evidence for thermal neutron values is lacking. Due to the low doses utilized for our 31 meV and 64 meV thermal neutron studies, direct evidence is provided for the respective DCA and micronucleus assay RBE_M_ values. It should be noted that the absorbed doses for the photon reference radiation dose-response curve and the thermal neutron dose-response curve were not perfectly matched and that extrapolation of the photon dose-response curve was required for this analysis.

Microdosimetry data demonstrated that higher doses resulted in an elevated probability of hits per cell. However, the probability of two or more hits per cell increased in a non-linear fashion, more so than single hits (Table 7). For example, for the maximum dose of the two irradiation configurations, the dose increased ~5-fold, whereas the probability of two or more hit increased nearly 20-fold.

In an effort to explain the RBE dose effect, Kellerer and Rossi previously proposed that neutron dose-effect relationships could be explained by a dual-hit theory [37]. This formalism relies on the assumption that there are no DNA lesion-modifying factors that vary with radiation type or energy distribution, which has been called into question [38]. However, the present study confirms the overall dose-dependent trend of the Kellerer and Rossi model as elevated RBE values were observed at lower doses.

### Radiation protection implications

The ^14^N(n,p)^14^C thermal neutron capture reaction contributed ~71% of the absorbed dose in both our 31 meV and 64 meV test systems [11]. Thermal neutron capture cross-sections vary considerably across the thermal neutron energy range. As such, the variability in incident thermal neutron energy and ^14^N concentration in a biological medium (e.g. 2.49% in soft tissue [9] or 5.70% in eye lens [39]) can dramatically affect biological detriment in small or shallow tissue targets (Supplement S1).

**Figure 8:**
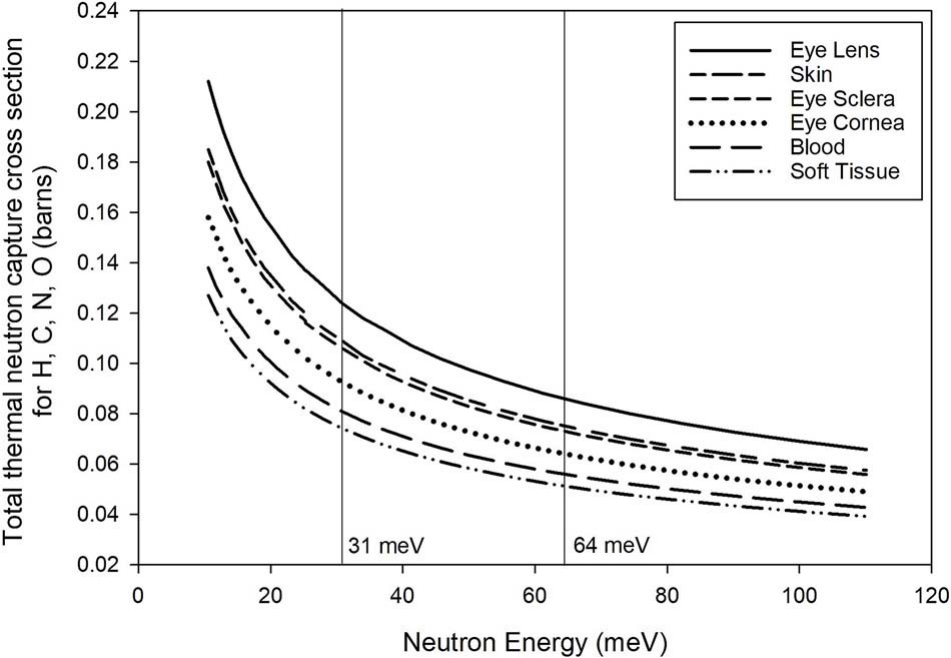
Total thermal neutron capture cross-sections for (n,p) plus (n,γ) in various shallow tissue targets. Cross-section data from IAEA ENDF B-VII.0 library [29]. Percent mass fraction inputs for individual tissues are presented in Supplement S1.

Radiation-induced cataractogenesis is of particular concern to individuals who receive notable radiation doses to the eye lens, including astronauts [40], radiologic technologists [41], computed tomography scan patients [42], and atomic bomb survivors [43]. Prior lens opacity studies in mice found RBE values from 3 to 10 following thermal neutron exposure range [44–46]. This supports our assumption of elevated RBE values in shallow targets. Furthermore, there is a case to be made that low doses of ionizing radiation in non-lens ocular tissues are involved in low-dose cataractogenesis [47]. Compared to blood, the eye lens, sclera, cornea, and skin all contain higher mass fractions of nitrogen, which has a higher thermal neutron cross-section than the other elemental constituents. The eye lens has the largest total thermal neutron cross-section across the energy range evaluated owing to the elevated nitrogen levels (Fig. 8). As a result, the eye lens, sclera, cornea, and skin may demonstrate elevated thermal neutron RBE values compared with normal tissue or blood as derived previously [11, 48]. Given our RBE evaluations, it appears that the ICRP thermal neutron *w*_R_ value of 2.5 underestimates the radiation detriment to shallow tissue targets including the eye lens, sclera, cornea, and skin. For radiation protection purposes, it would seem prudent to evaluate separate thermal neutron *w*_R_ values specifically for the eye and other small and shallow targets.

There are still many unanswered questions regarding the large RBE variation of 5–51 in the DCA thermal neutron literature (energy range: 25–64 meV, as stated in four of the six reports; Table 1) [15–19]. Given the results of this report that suggest neutron energy spectra and/or dose rate differences resulted in significantly different RBE values when all other experimental characteristics were kept consistent (Table 5), it is possible that these factors could account for some of the thermal neutron RBE variation seen in the literature. Additional sources of RBE variation such as cell culture protocols, experimental timing, microscopy scoring logic, and choice of reference radiation may be the subject of future work, the goal of which is to provide confident thermal neutron RBE values for radiation protection decision-making.

### Limitations

Blood from three of the five DCA donors and two of the three micronucleus donors were irradiated at the lowest 41 mGy dose point. This greatly reduced the number of cells available for scoring and decreased the accuracy at the lowest dose point. The cell numbers at all dose points were limited by the volume of blood available for the assays. Blood donors enrolled in the current study differed from the blood donors enrolled in the reference radiation studies.

## Conclusions

The DCA and the cytokinesis-block micronucleus assay demonstrated a linear dose-response following low-dose thermal neutron exposure with an average energy of 31 meV. The RBE_M_ values for the DCA and micronucleus assay were elevated beyond the current ICRP *w*_R_ value of 2.5, and significantly lower than the RBE_M_ values given in our prior RBE_M_ study, which employed identical methodology but utilized thermal neutrons with a lower dose rate and a higher average energy of 64 meV. RBE dose dependence was confirmed at low thermal neutron doses ranging from 41 to 408 mGy. Our RBE evaluations suggest that it may be necessary to consider separate *w*_R_ values for small and/or shallow tissue target thermal neutron exposures. These findings are of particular significance for shallow tissue targets such as the skin and the lens of the eye for which the current *w*_R_ may underestimate the radiation detriment from thermal neutrons. Furthermore, our experimental findings provide direct evidence that incident thermal neutron spectra and/or dose rates are an important source of RBE variation in small and shallow tissue targets. This may explain some of the thermal neutron DCA RBE variation in the literature, although other factors are under further investigation.

## Supplementary Material

Supplement_S1_NEW_ncae231

Supplement_S2_NEW_ncae231

Supplement_S3_ncae231

Supplement_S4_ncae231
