# Supplementary material for: Relative biological effectiveness of 31 meV thermal neutrons in peripheral blood lymphocytes
Source: Radiat Prot Dosimetry. 2025 Mar 10;201(4):297–313. doi: 10.1093/rpd/ncae231 (PMC11926985; doi:10.1093/rpd/ncae231)
Supplement: Supplement_S1_NEW_ncae231 [file supplement_s1_new_ncae231.pdf]

Supplement S1. Percent mass fraction of H, C, N, O, and Si in relevant tissues.

Tissue	H	C	N	O	Si	Total	Reference
Blood	10.19	10.00	2.96	75.94	0.0030	99.09	McConn Jr et al. (2011)
Eye lens	9.60	19.50	5.70	64.60	-	99.40	Behrens at al. (2009)
Eye sclera	10.00	14.60	4.50	70.60	-	99.70	Nogueira et al. (2011)
Eye cornea	10.16	12.62	3.69	73.14	-	99.61	Behrens at al. (2009)
Skin	10.06	22.83	4.64	61.90	-	99.43	McConn Jr et al. (2011)
Soft tissue	10.44	23.21	2.49	63.02	-	99.16	McConn Jr et al. (2011)

REFERENCES

McConn RJ Jr, Gesh CJ, Pagh RT. et al. Compendium of material composition data for radiation transport modeling revision 1 (PIET-43741-TM-963, PNNL-15870 Rev. 1). 1-357 (2011).

Behrens R, Dietze G, Zankl M. Dose conversion coefficients for electron exposure of the human eye lens. Phys Med Biol 2009;54:4069–87. https://doi.org/10.1088/0031-9155/54/13/008.

Nogueira P, Zankl M, Schlattl H. et al. Dose conversion coefficients for monoenergetic electrons incident on a realistic human eye model with different lens cell populations. Phys Med Biol 2011;56:6919–34. https://doi.org/10.1088/0031-9155/56/21/010.
