# Supplementary material for: Relative biological effectiveness of 31 meV thermal neutrons in peripheral blood lymphocytes
Source: Radiat Prot Dosimetry. 2025 Mar 10;201(4):297–313. doi: 10.1093/rpd/ncae231 (PMC11926985; doi:10.1093/rpd/ncae231)
Supplement: Supplement_S2_NEW_ncae231 [file supplement_s2_new_ncae231.pdf]

Supplement S2. DCA scoring data by donor.

Total dose (mGy)	Cells scored	Aberr. Type			Total Dic. + Ring	Cellular distribution of Dic + Ring aberr					Aberr. per cell	Disp. index (σ^2/y)	*u*-test
		Dic.	Ring	Ace.		0	1	2	3	4			
DONOR A													
0	500	1	0	6	1	499	1	0	0	0	0.0020	1.00	-
41	122	0	0	1	0	122	0	0	0	0	0.0000	-	-
102	500	16	0	31	16	485	14	1	0	0	0.0320	1.10	1.55
204	118	13	0	14	13	106	11	1	0	0	0.1102	1.05	0.42
306	500	42	4	79	46	461	33	5	1	0	0.0920	1.26	4.13
408	500	85	9	157	94	423	62	13	2	0	0.1880	1.22	3.47
DONOR B													
0	213	0	0	3	0	213	0	0	0	0	0.0000	-	-
102	452	18	1	35	19	436	13	3	0	0	0.0420	1.28	-7.35
204	500	41	8	94	49	455	41	4	0	0	0.0980	1.07	1.08
306	500	68	9	120	77	431	62	6	1	0	0.1540	1.08	1.30
408	500	72	18	150	90	421	70	7	2	0	0.1800	1.11	1.76
DONOR C													
0	593	1	0	8	1	592	1	0	0	0	0.0017	1.00	-
41	500	8	3	25	11	491	7	2	0	0	0.0220	1.34	5.70
102	500	23	4	51	27	477	19	4	0	0	0.0540	1.24	3.94
204	500	37	3	68	40	462	36	2	0	0	0.0800	1.02	0.35
306	573	78	8	140	86	497	68	6	2	0	0.1501	1.13	2.23
408	387	81	8	143	89	314	60	11	1	1	0.2300	1.22	3.11
DONOR D													
0	420	0	0	4	0	420	0	0	0	0	0.0000	-	-
102	265	4	1	10	5	260	5	0	0	0	0.0189	0.98	-0.19
204	500	34	7	77	41	463	33	4	0	0	0.0820	1.12	1.84
306	500	80	5	136	85	427	63	8	2	0	0.1700	1.16	2.57
408	500	101	8	141	109	412	69	17	2	0	0.2180	1.21	3.28
DONOR E													
0	600	0	0	2	0	600	0	0	0	0	0.0000	-	-
41	366	9	0	17	9	359	6	0	1	0	0.0246	1.65	9.26
102	500	27	1	45	28	476	20	4	0	0	0.0560	1.23	3.73
204	500	43	4	61	47	460	34	5	1	0	0.0940	1.25	3.97
306	534	72	3	116	75	471	52	10	1	0	0.1404	1.21	3.43
408	500	100	6	175	106	414	66	20	0	0	0.2120	1.17	2.66

Aberr., aberrations; Dic., dicentric chromosome; Ace. Acentric chromosome; Disp., dispersion.
