# Supplementary material for: Relative biological effectiveness of 31 meV thermal neutrons in peripheral blood lymphocytes
Source: Radiat Prot Dosimetry. 2025 Mar 10;201(4):297–313. doi: 10.1093/rpd/ncae231 (PMC11926985; doi:10.1093/rpd/ncae231)
Supplement: Supplement_S3_ncae231 [file supplement_s3_ncae231.pdf]

Supplement S3. Curve fitting for individual donors.

Dicentric Chromosome Assay							
Donor	Regression	Equation ($A = c + \alpha D$)	α [±SE] (Gy^{-1})	c [±SE]	χ^2-test sig.	α z-test sig.	Pearson's R^2 value
A	Linear	$A = 0.001 + 0.382D$	0.382 ± 0.049	0.001 ± 0.005	0.517	0.002	0.95
B	Linear	$A = 0.465D$	0.465 ± 0.030	-	0.786	< 0.001	0.99
C	Linear	$A = 0.002 + 0.492D$	0.492 ± 0.032	0.002 ± 0.004	0.710	< 0.001	0.99
D	Linear	$A = 0.494D$	0.494 ± 0.056	-	0.032	0.003	0.98
E	Linear	$A = 0.495D$	0.495 ± 0.030	-	0.909	< 0.001	1.0
Micronucleus Assay							
Radiation	Regression	Equation ($A = c + \alpha D + \beta D^2$)	α [±SE] (Gy^{-1})	c [±SE]	χ^2-test sig.	α z-test sig.	Pearson's R^2 value
B	Linear	$A = 0.010 + 0.303D$	0.303 ± 0.080	0.010 ± 0.019	-	0.032	0.90
C	Linear	$A = 0.012 + 0.136D$	0.136 ± 0.040	0.012 ± 0.009	0.500	0.028	0.87
E	Linear	$A = 0.023 + 0.371D$	0.371 ± 0.075	0.023 ± 0.023	-	0.008	0.91

A, Aberrations per cell; *α, β, c*, regression coefficients; *D*, Dose (Gy); R^2, coefficient of determination; Sig., Significance.
