# Supplementary material for: Relative biological effectiveness of 31 meV thermal neutrons in peripheral blood lymphocytes
Source: Radiat Prot Dosimetry. 2025 Mar 10;201(4):297–313. doi: 10.1093/rpd/ncae231 (PMC11926985; doi:10.1093/rpd/ncae231)
Supplement: Supplement_S4_ncae231 [file supplement_s4_ncae231.pdf]

Supplement S4. Micronucleus data by donor.

Total dose (mGy)	BNCs scored	Total MN	Cellular distribution of MN				Total MN Per Cell	Disp. index (σ^2/y)	*u*-test
			0	1	2	3			
DONOR B									
0	1714	22	1694	18	2		0.013	1.17	5.08
102	1000	34	966	34			0.034	0.97	-0.75
204	1613	80	1540	66	7		0.050	1.13	3.60
306	807	61	753	47	7		0.076	1.16	3.14
408	1000	195	841	126	30	3	0.195	1.21	4.62
DONOR C									
0	2920	27	2893	27			0.009	0.99	-0.35
41	1000	34	970	26	4		0.034	1.20	4.59
102	826	14	813	12	1		0.017	1.13	2.68
204	924	37	889	33	2		0.040	1.07	1.51
306	1000	39	962	37	1		0.039	1.01	0.30
408	817	66	759	50	8		0.081	1.16	3.32
DONOR E									
0	1000	33	969	29	2		0.033	1.09	2.03
41	2000	61	1943	54	2	1	0.031	1.13	4.27
102	1000	67	943	48	8	1	0.067	1.26	5.91
204	1000	44	960	37	2	1	0.044	1.18	4.17
306	2000	323	1733	216	46	5	0.162	1.22	6.87
408	1386	243	1196	139	49	2	0.175	1.28	7.34

BNCs, bi-nucleated cells, MN, micronuclei, Disp., dispersion.
